# Worldwide Research Trends on Solar-Driven Water Disinfection

**DOI:** 10.3390/ijerph18179396

**Published:** 2021-09-06

**Authors:** Menta Ballesteros, Celeste Brindley, José Antonio Sánchez-Pérez, Pilar Fernández-Ibañez

**Affiliations:** 1 Molecular Biology and Biochemical Engineering Department, Experimental Sciences Faculty, Universidad Pablo de Olavide, Ctra. de Utrera km 1, 41013 Seville, Spain; 2 Department of Chemical Engineering, Universidad de Almería, 04120 Almería, Spain; cbrindle@ual.es (C.B.); jsanchez@ual.es (J.A.S.-P.); 3 CIESOL, Joint Center of the Universidad de Almería-CIEMAT, 04120 Almería, Spain; 4 Nanotechnology and Integrated BioEngineering Centre, School of Engineering, Ulster University, Newtownabbey BT37 0QB, UK

**Keywords:** disinfection, solar, water recycling, photocatalysis, pathogens, inactivation

## Abstract

“Ensure access to water for all”, states Goal 6 of the UN’s Sustainable Development Goals. This worldwide challenge requires identifying the best water disinfection method for each scenario. Traditional methods have limitations, which include low effectiveness towards certain pathogens and the formation of disinfection byproducts. Solar-driven methods, such as solar water disinfection (SODIS) or solar photocatalysis, are novel, effective, and financially and environmentally sustainable alternatives. We have conducted a critical study of publications in the field of water disinfection using solar energy and, hereby, present the first bibliometric analysis of scientific literature from Elsevier’s Scopus database within the last 20 years. Results show that in this area of growing interest USA, Spain, and China are the most productive countries in terms of publishing, yet Europe hosts the most highly recognized research groups, i.e., Spain, Switzerland, Ireland, and UK. We have also reviewed the journals in which researchers mostly publish and, using a systematic approach to determine the actual research trends and gaps, we have analyzed the capacity of these publications to answer key research questions, pinpointing six clusters of keywords in relation to the main research challenges, open areas, and new applications that lie ahead. Most publications focused on SODIS and photocatalytic nanomaterials, while a limited number focused on ensuring adequate water disinfection levels, testing regulated microbial indicators and emerging pathogens, and real-world applications, which include complex matrices, large scale processes, and exhaustive cost evaluation.

## 1. Introduction

Access to safe drinking water is a global concern with implications to public health and well-being and to global economic development. Water scarcity affects over 40% of the global population and is expected to rise over the next years, in which, by 2025, half of the world’s population will live in areas that suffer from water shortage [1]. According to the United Nations’ 2030 agenda, the Sustainable Development Goal 6 (SDG6) aims to achieve universal and equitable access to safe and affordable drinking water for all [2]. This remains a huge challenge for low-income countries, where 3 out of 10 people lack access to safely managed drinking-water services. It is estimated that water-related issues cause 485,000 deaths by diarrhea each year [3]. These vulnerabilities become worse in the case of natural or man-made disasters such as global climate change and the Covid-19 pandemic; according to the UN, stopping the pandemic requires accessibility to safe water for people living in these areas [2]. 

Water pollution—with over 80% of the wastewater (WW) resulting from human activities being discharged without any treatment—and overexploitation of the freshwater resources— as approximately 70% of global withdrawals from rivers, lakes and aquifers are used for irrigation—are the main issues in developing countries. Worldwide, China and India are at the top of the list of countries with the highest water footprints, followed by the USA. The SDG6′s aim is “to improve water quality by reducing pollution, halving the proportion of untreated wastewater, and substantially increasing recycling and safe reuse globally” [4]. Therefore, the use of non-conventional sources of water, including WW recovery technologies and rain and storm water treatment, has become an increasingly important strategy to offset scarcity. In this context, disinfection of WW effluents or any other non-conventional freshwater sources is key to producing biorisk-free freshwater resources and avoid undesirable public health and food safety issues.

Disinfection byproducts (DBP)—many of them potentially mutagenic and carcinogenic—are generated when disinfectants react with naturally occurring organic matter and other compounds in water, and their presence in drinking water is the main drawback of chemical disinfection [5]. Furthermore, the use of these disinfectants is connected to a high level of microbial resistance, which can involve re-growth of resistant pathogens after disinfection [6]. Likewise, the World Health Organization (WHO) has identified the infections by pathogens that are resistant to antimicrobials, including antibiotic-resistant bacteria (ARB), as one of the major risks for global health. The problem is aggravated if the pathogenic bacteria that bear antibiotic-resistant genes (ARG) pass on this resistance trait, through the ARG, to other bacteria in the water, thus preserving the antimicrobial property even after disinfection [7]. Therefore, new methods for water disinfection that are effective against these emerging pathogens as well as being affordable and environmentally sustainable are very necessary.

This is the case of solar water disinfection (SODIS), which was recognized by the WHO as a simple, effective methodology for treating water polluted with pathogenic microorganisms and for reducing the incidence of diarrheic diseases through solar exposure [8,9]. The SODIS technique consists of placing raw water in transparent, plastic containers (usually 1-to 2-L PET bottles) and exposing them to direct solar light for at least 6 h (on a cloudy day the exposure time should be increased to 48–72 h); afterwards, the water should be stored in the SODIS bottles and drunk within the next 24 h [10]. The mechanisms that explain the germicidal effect of SODIS are based on the generation of reactive oxygen species upon the absorption of UVA and UVB photons by chromophores in the microorganisms; the efficiency of the process is improved by increased temperatures due to solar exposure [11].

SODIS is particularly interesting from both economic and environmental viewpoints for several reasons: it has low operating costs for the user (solely based on the replacement of the bottles), it is very simple to operate and uses only sunlight, and it has no effect on the organoleptic properties of the water, nor requires additional chemicals nor generates any residues. In addition, SODIS has been demonstrated to be effective against several water pathogens, i.e., *Escherichia coli, Salmonella spp., Vibrio cholerae, Enterococcus faecalis*, Bacteriophage MS2, *Hepatitis A virus*, and *Cryptosporidium parvum* [12]. However, it has several drawbacks that have been the subject of recent studies for their mitigation: (i) The water treated with SODIS, as mentioned, must be drunk within 24 h to avoid the re-emergence of pathogenic organisms’ activity after solar exposure [13]; (ii) The action of sunlight on the plastic material may release chemical products into the drinking water. There are controversial studies about the detection and measurement of photoproducts from the PET containers: some suggest that these derivates do not exceed the limits established for the quality of drinking water yet do exceed the limits in bags manufactured with the same material [14], while others determine that the emitted products are mostly volatile and do not accumulate in the water at sufficient concentrations to raise health concerns during the daily use of the technique, and they have been proven to produce a non-significant increase in the genotoxicity of the treated water [15]; (iii) The volumes treated were limited (containers were frequently of no more than 2 L, as pointed out previously), but in recent years some alternative, viable strategies have been demonstrated, such as the use of solar reactors, bags and bottles of larger capacities, capable of treating up to 200 L per day [16,17]; (iv) Incoming solar UV-radiation is limited, although it can be increased through the use of solar mirrors, including compound parabolic collectors among others [16,18].

Photocatalysis emerged at the end of the past century as an alternative or complementary technology to traditional disinfection methods. It is divided into heterogeneous and homogeneous processes, which are part of the advanced oxidation processes (AOPs), and in both cases sunlight can be used to promote the formation of hydroxyl radicals (^●^OH), which exhibit a strong disinfectant activity [19]. During heterogeneous photocatalysis, the inactivation of pathogens in water occurs in the presence of a photocatalyst and UV radiation. Although many catalysts have been tested, in disinfection processes TiO_2_ is the most studied [20]. However, when sunlight is used as an energy source in heterogeneous photocatalysis the activity of the process is low because photocatalysts are only excited by UV radiation and the proportion of this type of radiation in sunlight is low (3–5%). The process is much more efficient when the catalyst is in the form of suspended micro- or nanoparticles, which can be immobilized to avoid contamination by nanoparticles [21], although immobilization limits the large-scale applications compared to homogeneous processes. In homogeneous solar photocatalysis the most investigated process is solar photo-Fenton treatment, which consists of a series of reactions involving solute iron acting as the catalyst, hydrogen peroxide and solar radiation [22]. Pathogens that are highly resistant to conventional disinfection technologies can be inactivated by solar photo-Fenton such as *Bacilus subtillis*, phytopathogenic fungi, antimicrobial resistant bacteria and genes, as well as viruses from surface water and WW [23,24,25,26,27]. At present, this field research is focused on limiting the pH of the process in the search for good results at neutral or near-to-neutral pH, assessing the effect of organic matter or of using iron oxides, iron-chelating agents, etc., to retain the iron in solution [28]. In addition, in recent years intensive research has been carried out under continuous mode operation, which shows the possibility of applying this technology at an industrial scale [29,30].

Table 1 presents a summary of the main events, breakthroughs, and contributors in relation to SODIS and water disinfection by solar photocatalysis.

Because of the many open paths of research that have been proved effective for pathogen inactivation in water and use sunlight as the only driving force, the state of the art in this area must be revised. The aim of this work is to identify the key research topics, research gaps and main challenges that allow establishing future lines of work in this area. With this objective in mind, we have conducted an ample, bibliometric study, in which we have made a critical and detailed assessment of the scientific production on water disinfection driven by solar radiation. Finally, based on an in-depth discussion of the results, we have attempted to answer the following research questions (RQs), which we believe could be of interest to researchers in this field:

RQ1: What are the global trends of scientific publications on the topic of solar-driven water disinfection?
RQ2: Which are the institutions and their collaboration networks that work more intensely on this issue?
RQ3: Which peer-reviewed journals publish the most on this topic?
RQ4: How has the field evolved over time and what are its main, future research directions?

## 2. Materials and Methods

The data analyzed in this work have been obtained mainly from the databases Scopus and, to a lesser extent, the Web of Science (WoS). Both bibliographic databases are the main sources of information used in bibliometric studies [40,41] because of the large number of documents they contain. The number of peer-reviewed journals indexed in Scopus (41,462; SCImago Journal Rank, 2019) is almost double to that of WOS (24,717; Thomson Reuters, 2019). In our case, WOS was used to search for impact indexes, whereas an exhaustive search was carried out in Scopus using (TITLE-ABS-KEY (Solar and Disinfection)) as the search field. This search returned 1241 documents between 1977 and 2020. Frequency of co-occurrences was calculated obtaining 533 keywords repeated more than 10 times. Note that the occurrences attribute indicates the number of documents in which a keyword occurs. When analyzing the keywords, those that had no relation with the topic of this study were manually removed, such as article, paper, review, etc. Quantitative data processing, positioning and visualization of the corresponding units of analysis in two-dimensional maps were carried out with VOSviewer software (Centre for Science and Technology Studies, Leiden University, Leiden, The Netherlands) using the VOS (visualization of similarities) technique, which builds a similarity matrix from the co-occurrence matrix to allow the execution of a clustering algorithm for positioning and classifying the keywords to be mapped. This software, widely used in bibliometric studies, creates graphs for keywords or institutions, representing them with nodes that can be joined by lines that signify the collaboration between both terms of the two nodes [42].

Hereby, a bibliometric map with 6 clusters was obtained. Cluster size is determined by the number of keywords within the cluster, the frequency of occurrences of the keywords and their similarity index. In this type of map, keywords from clusters located in the center of the map are greatly interrelated, unlike those from clusters that are further away. Finally, a color scale map was obtained considering the frequency of keywords according to the year of publication within the last decade. In both maps the size of the label is proportional to the keyword’s frequency of appearance within the documents. It should be noted that, while all representative terms are in display, many of the 533 keywords, generally subordinate terms, are not, for the sake of clear visualization.

## 3. Results

### 3.1. Evolution of the Scientific Production

**Figure 1:**
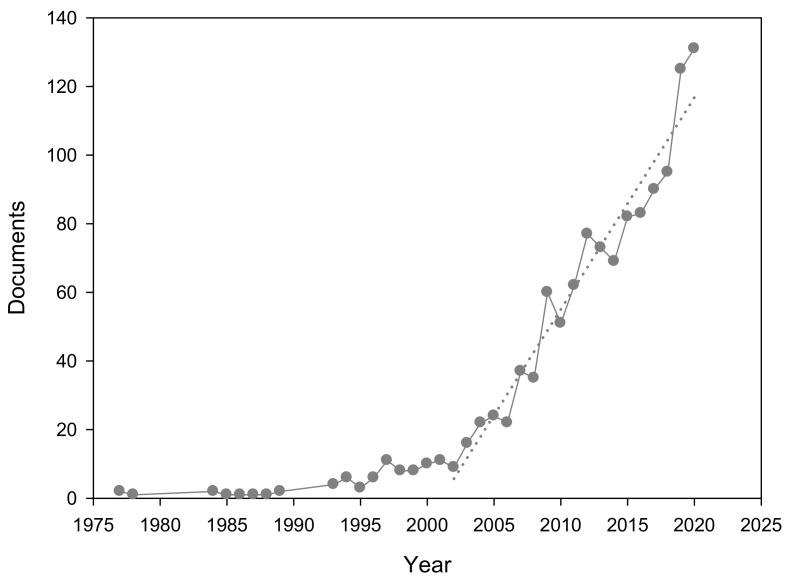
Number of documents in the field of research on solar-driven disinfection from 1977 to 2020.

The search process returned 1241 documents from first records to 2020, showing a two-speed evolution (Figure 1). From the first publication in 1977 to 2002, the number of publications in this field was very scarce, with an average of less than 5 documents per year. However, from 2002 to 2020 a linear growth is observed, showing the rapidly growing relevance of this research field, with rising numbers of publications each year (increasing by over 10-fold the number of publications in this period, going from only 9 in 2002 to 131 in 2020). As shown in Figure S1 (Supplementary Material), most of the publications on solar-driven disinfection are articles (77%) and it should be noted that, of all those publications, only approximately 20% have been published ‘open access’ in the last 10 years (Figure S2; Supplementary Material).

### 3.2. Distribution of Publications by Origin

**Figure 2:**
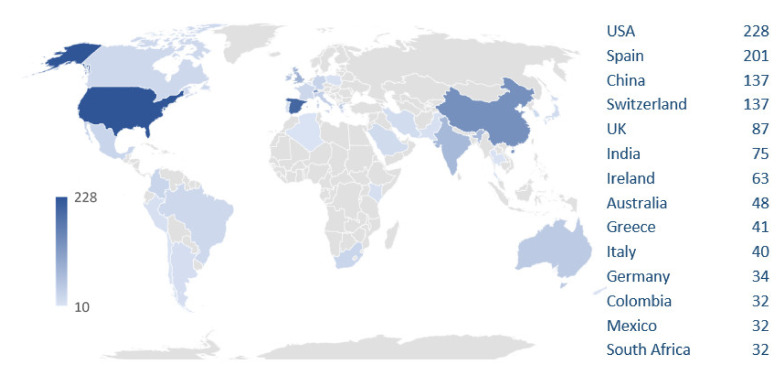
Distribution of publications on solar-driven disinfection in countries with more than 10 publications (Powered by Bing © GeoNames, Microsoft, Navinfo, TomTom, Wikipedia).

Worldwide, distribution is very diverse in terms of the number of publications that are spreading over every continent and country (Figure 2), highlighting the close collaboration between most of them, as can be seen in Figure S3 (Supplementary Material), showing the relationships between the different countries according to joint publications. Although the USA stands out for having the largest number of publications, with a total of 228 (Figure 2), these are widely distributed among various institutions and authors (see Table 2, which shows the total number of documents per affiliation and per author of the most prolific centers). A very different scenario is observed in Spain, second in the lead in total number of publications with 201 documents, where the Center for Energy, Environmental and Technological Research (CIEMAT)-Plataforma Solar of Almería concentrates most of the publications. Next, the Ecole Polytechnique Fédérale de Lausanne (Switzerland) stands out on a European level. China is also the third potential in the world (along with Switzerland) on solar-driven disinfection (Figure 2), with a total of 137 publications, and as with the USA, its production is spread among many research centers and authors. In terms of the number of investigations that receive funding, however, this country is highest in the list (Table S1; Supplementary Material), but if all the research carried out within the European continent is considered as well as the research funded by the European Union and the research from Spain and Switzerland, Europe would be world leader with the financing of a total of 128 publications. The USA also ranks highly, with funding for a total of 29 publications.

### 3.3. Journals of Greater Impact

**Figure 3:**
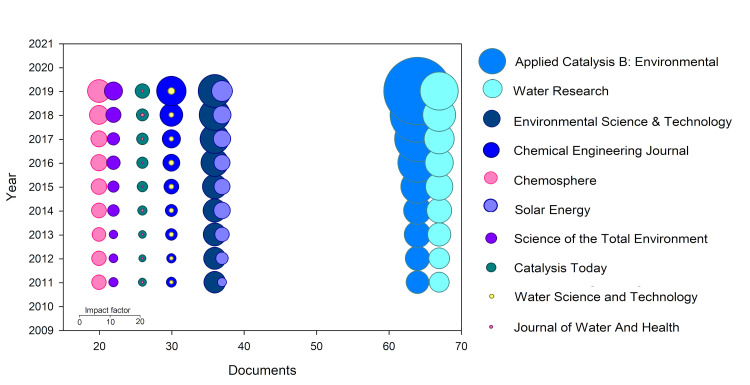
Journals with the highest number of articles on solar-driven disinfection and evolution of the impact index over the last 10 years for the journals with the highest number of documents. The size of the circles represents the impact factor according to the scale given.

The journals with the highest numbers of publications are *Applied Catalysis B: Environmental* and *Water Research*, with 64 and 67 articles, respectively (Figure 3). Both journals have evolved exhibiting positive growth over the last decade (Figure 3), especially in the case of *Applied Catalysis B: Environmental* whose impact factor increased very significantly, tripling its value from 2011 to 2019 (currently with an IF of 16.686). *Chemical Engineering Journal* is in second position according to its IF in 2019 (IF = 10.652), with a very prominent number of documents (36) on solar-driven disinfection, followed by *Water Research* (IF = 9.130) and *Environmental Science & Technology* (IF = 7.864), (Figure 3). All these journals are in the first quartile of the categories included in Journal Citation Reports and have a great impact on scientific society. Furthermore, *Applied Catalysis B* and *Chemical Engineering Journal* are in the first decile in Engineering Environmental category (1/53 and 2/53, respectively), and *Water Research* and *Environmental Science & Technology* are also positioned in the first decile in Water Resources (1/94) and Environmental Sciences (15/265) categories, respectively. The fifth place is for *Solar Energy* that, although with a lower impact index, has been able to double its value in the period 2011–2019, highlighting the growing relevance of solar technologies research.

### 3.4. Analysis of the Keywords

So far, Scopus’s search export has been processed to analyze the number of publications by year, their distribution by affiliation and country, the most prolific institutions, and the most relevant journals and authors. Next, the keywords used in the scientific literature will be analyzed to identify the research trends and gaps in the field.

**Figure 4:**
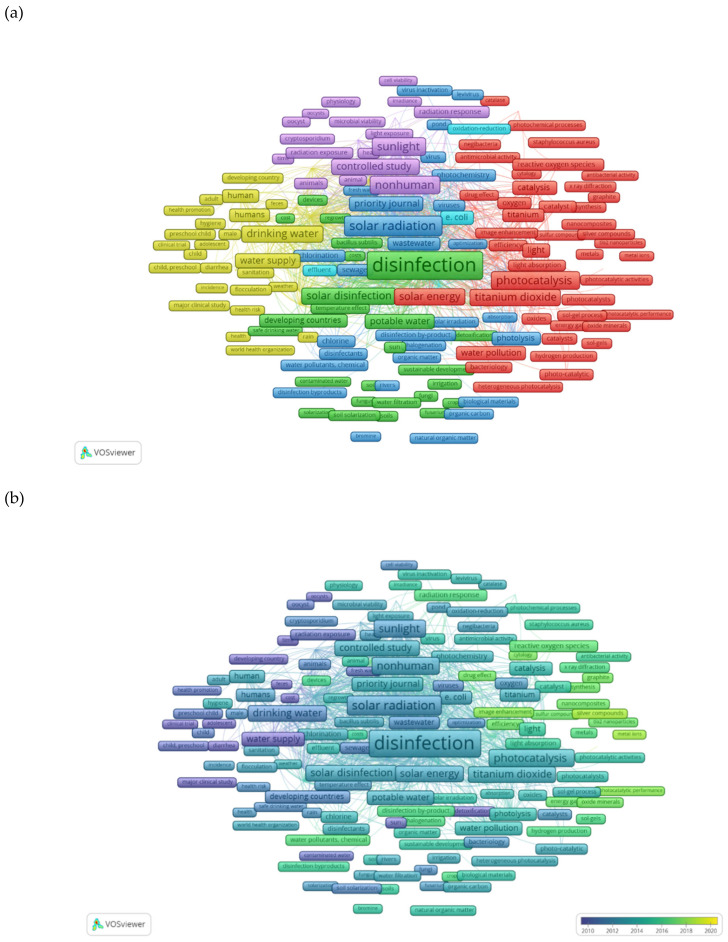
Clusters of keywords that appear in articles about solar-driven disinfection with a frequency greater than 10: (**a**) up to the year 2020; (**b**) in the period 2010–2020, where the color scale indicates the year of publication, with colors ranging from blue for the lowest score (2010) to green (intermediate years) and to yellow for the highest score (2020). Note that, while all representative terms are in display, many of the 533 keywords are hidden, for the sake of clear visualization.

Figure 4 represents the network formed by the main keywords from publications on solar-driven disinfection. To discuss these results, only keywords with concurrency greater than 10 have been considered, but for better understanding only those with greater repeatability are shown in Figure 4 (complete keywords’ matrix available from Supplementary Materials: File S1). Each rectangle-shaped node represents a keyword, and the larger the rectangle the more frequent the keyword. In Figure 4a, keywords are classified into six clusters using VOSviewer, and each cluster has a different color attending to the relations established among keywords (characterized by the frequency of occurrences and the similarity index). Nodes are connected by lines that join terms that tend to appear in the same work, broad lines standing for a more frequent coexistence than thin lines. Regarding the location of the clusters in the network, the closer they are to each other the stronger their relatedness. The evolution of the relevance of the keywords over the last decade (2010–2020) is depicted in Figure 4b, in which keywords are mapped using a color scale that assigns each node with a score (in this case, the scores are years 2010 to 2020). To interpret this figure, it should be noted that the most frequent terms closer to 2010 are in blue (lowest score), intermediate scores are in green, and scores closer to 2020 are mapped in yellow.

#### 3.4.1. Green Cluster

This is the centermost cluster, the one closest to the rest of the clusters and the second in size (Figure 4a). It is formed by a total of 137 keywords including *disinfection*, *solar disinfection*, *water treatment*, *WW reclamation*, *WW recycling*, *potable water*, *detoxification* and similar terms focusing on *irrigation* (*agriculture*), *crop*, *soil solarization* in *groundwater* and *natural water*, organisms such as *fungi*, specifically, *Fusarium solani* and *Lycopersicon esculentum*, *Bacillus subtilis*, *nematodes*, and places such as *India*, *Spain* or the *USA*. This set of keywords is related to one of the areas of greatest interest to the field of disinfection of water for agricultural uses, which is the largest water-consuming sector (approximately 70% of the total, as previously pointed out). Water for agricultural use very frequently has phytopathogens and it is common to use fungicides and antagonistic microorganisms to avoid this problem. Many of the articles that describe the use of solar energy in disinfection have shown good results in the inactivation of phytopathogens [23]. Even the mere sunlight-H_2_O_2_ combination has recently been considered as a sustainable option, compared to other solar-powered AOPs in advanced municipal wastewater treatment plants (WWTPs), for reuse in crop irrigation [43]. Recent articles clearly show the current research trends in this area, including the investigation of microbial risks associated to the practice of WW reclaim in agriculture [44], the implementation of large-scale continuous solar treatments [29] and the control of emerging pathogens, such as antimicrobial resistance for irrigation and food production [45].

One of the limitations of disinfection is the persistence of the treatment once applied, hence the importance of the keyword *regrowth/bacterial regrowth* that appears in this cluster. In fact, it is known that in many cases microorganisms can reactivate or repair themselves from photo-induced DNA damage and some surviving microorganisms can even reproduce [22]. Likewise, according to the review carried out by Wang et al., (2021), regrowth studies should be carried out simulating actual conditions, considering factors such as organic and inorganic matter or light/dark conditions, and carrying out multiple suitable detection methods when performing microbial counts.

#### 3.4.2. Dark Blue Cluster

As can be seen in Figure 4a, this is a transversal cluster and, similarly to the green cluster, its keywords are distributed at the center of the map and very close to the other clusters, but especially intermingled with the green and light blue clusters, so it is not considered a specific area of research. It includes keywords such as *solar radiation*, *photochemistry*, *chlorination*, etc. An outstanding area to consider in disinfection studies is the presence of *disinfection byproducts* (DBPs), hence keywords such as *chlorination* or *chlorine*. Solar-driven disinfection processes have become particularly important in this area because, although the lack of a residual disinfection effect is a disadvantage, these processes do not generate any harmful byproducts and are environmentally friendly. Moreover, it has been widely demonstrated that these processes are efficient in eliminating DBPs generated by other classic water disinfection processes [6]. It is also well known that organic matter reduces the efficiency of most AOPs, although it can also help maintain the iron catalyst in solution when pH-neutral reactions are carried out [46,47]. NOM has also been found to favor SODIS under specific conditions—photosensitization effects—but it also can compete with microorganisms for the oxidizing radicals generated during photo-assisted processes, hence decreasing the disinfection efficiency [48].

The reoccurrence of the keywords *virus, levivirus*, and *virology* is particularly striking and focuses attention on these types of special organism since, in comparison with bacterial pathogens, viruses have a lower infection dose and a higher risk of disease, which means that they pose a serious health threat. Furthermore, in this area it is advisable to use surrogates (e.g., bacteriophages) of pathogenic viruses capable of causing human disease to test out photocatalytic viral inactivation processes and mechanisms [49]. Current research shows a clear trend towards the control of highly resilient, emerging, waterborne pathogens, such as new human viruses, and antimicrobial resistance—ARB and ARG- in water.

The keyword *compound parabolic collectors* (CPCs) appears in this cluster because these systems have been intensively investigated as a modular way to scale-up the disinfection process with high efficiency for the harvest of solar radiation. However, to treat the volumes required for real applications (a few hundred liters per day per household, for drinking water, and a few cubic meters per day, for irrigation) new solar raceway pond reactors operated continuously appear as a good solution [29], despite having lower efficiency optics compared to CPCs. This is evidenced by the also present keyword *pond* as, in recent years, ponds have become a class of successful and practical reactors that clearly allow increasing the treatment volume of SODIS. Currently, solar photocatalysis, photo-Fenton and solar disinfection have been taking steps in the right direction to expand promising results at these large scales [29,50,51]. Additionally, the integration of technologies based on photocatalytic and photothermal nanomaterials to increase the daily productivity of SODIS has been proved recently [34]. Other types of reactors have also been used, such as the photocatalytic membrane reactor, which integrates photocatalysis and membrane separation, photoelectrocatalytical reactors [49], etc. However, most of this technology is designed for water decontamination but not specifically focused on disinfection as the main objective, as explained by Keane and collaborators [39]. These authors recommend that efforts should focus on specifically designing efficient reactors for disinfection purposes, taking into account key technological parameters related to design of solar CPC modules (ratio of illuminated volume to total volume, catalyst load in slurry reactors, immobilized versus suspended photocatalyst, and flow rate), oxygen transfer kinetics, mass transfer of bacteria to the catalyst, and the catalyst support configuration.

The keyword *cost* appears repeatedly in several clusters because many of them refer to the fact that these are low-cost technologies because they use sunlight and are not dependent on fossil fuels. Especially cheap is SODIS technology, which is considered the lowest-price household-based disinfectant system, with an estimated cost of US$0.63 per person per year [52]. However, there is a great paucity of research regarding the true cost of photocatalytic disinfection treatments. A recent study (Fe^3+^-EDDHA/H_2_O_2_/solar treatment) carried out with compound parabolic collector reactors estimated the cost of the treatment (EUR 1.10 €/m^3^) as a sum of the investment and operational costs based on the calculation of their annual cost. The authors concluded that the estimated treatment cost is too high for industrial-scale applications because some techno-economic aspects are still unclear [53]. Each investigation must be considered in its own context: the first study mentioned here—SODIS in PET bottles—presented estimates for household interventions to provide safe drinking water, while the second—photo-Fenton—referred to large-scale treatment of wastewater for irrigation reuse; two completely different applications and end-users. Treatment costs must be kept in mind, especially in countries with populations that have limited access to potable water and sanitation. For example, in India (the worst country in the world for the number of people without safe water) the cost of 50 L of water is 17% of a typical poor person’s salary [54].

#### 3.4.3. Light Blue Cluster

This cluster mainly contains articles on AOP treatments, including the following keywords: *Fenton, photo-Fenton,* the implicit use of *hydrogen peroxide, UVC radiation,* and processes of *oxidation-reduction.* It also includes *E. coli* or *coliform* and *Enterococcus faecalis,* which are used as model organisms or indicators of water quality in microbiological terms. *E. coli* is a gram-negative coliform bacterium that is responsible for childhood diarrhea and is used as an indicator of fecal contamination, so it is widespread in disinfection articles. However, the WHO has recommended monitoring the gram-positive bacterium *E. faecalis* as an indicator of fecal contamination in water, and it is usually more resistant than *E. coli* to the different disinfection techniques [49]. It is important to note that each country has different indicators, but there is a consensus between organizations such as WHO, US EPA, etc., over which species should be studied to ensure water disinfection. Therefore, studies that arbitrarily employ other infective agents may even be of little relevance, depending on which microorganism they use.

#### 3.4.4. Yellow Cluster

This cluster contains 59 keywords, including *drinking water* and *water supply*, *water purification, water management,* and *water microbiology*. This cluster focuses on the purification of water for drinking purposes, including in this category the distinction between *female* and *male*, as well as adults, adolescent, infant or *child* and *preschool child*. It is known that gender roles need to be taken into consideration for interventions such as SODIS to be successful [55], and adverse effects observed from some toxic compounds in water are highly dependent on gender [56]. However, we have not detected any studies that address the inclusion of gender perspective into the investigation of water disinfection mediated by solar radiation.

This cluster also focuses on the purification of *rainwater* using *sunlight* as source of energy in *rural areas* or *developing countries*, including studies in *Kenya* or Bolivia, and is centered on the issue of public health, including the reduction of *diarrhea* through the inactivation of *fecal coliform* and *Vibrio cholerae*. All keywords clearly imply an area of research into SODIS, whose concordance is also evident in the closeness of keywords such as *SODIS, plastic* (referring to the PET bottles commonly used to carry out SODIS disinfection), or *disease control*.

#### 3.4.5. Purple Cluster

This cluster includes the words *nonhuman*, *sunlight,* and *controlled study*, among others, within investigations associated to disinfection of the pathogen *Cryptosporidium.* These keywords highlight an important area in relation to the inactivation of this enteric pathogen for vertebrates, recognized as a cause of disease in humans and domestic animals for over 50 years (hence the appearance of the terms *oocyst* or *protozoa*) and that can be inactivated using SODIS and solar photocatalytic disinfection [57] or a combination of TiO_2_ and H_2_O_2_, and recently with photo-Fenton processes [58].

In this cluster, *radiation/light exposure* or *radiation response* and *time* also stand out, referring to the well-known proportional relationship between the energy supplied and the degree of disinfection achieved. Treatment duration is mainly dependent on the nature of the microorganism, although water matrix, temperature, and wavelength play important roles [22,59]. It is well known that *Cryptosoridium* is one of the highest solar-resilient water pathogens and for this reason these keywords appear together.

#### 3.4.6. Red Cluster

This is the largest cluster, which includes 148 items or keywords. Furthermore, it is also the most recent and the most studied cluster (Figure 4b), which shows that the current trend is to focus more on solar photocatalysis with TiO_2_. Typical keywords of this process are *photocatalysis/catalyst/heterogeneous photocatalysis,* and *titanium/titanium dioxide/TiO_2_*. Much of the research conducted to date has been applied to the inactivation of *E. coli*, the most salient keyword. Furthermore, many words also feature very prominently in relation to nanoparticles, such as *nanocomposite, nanomaterial, metal nanoparticle*, *silver nanoparticle*, etc. There is a great diversity of TiO_2_-doped nanomaterials with metals and non-metals, such as nitrogen, silver, copper, sulfur, carbon, etc., and recently graphene, used to inactivate various strains of bacteria in different sources of water [20]. These doped materials and the combination of TiO_2_ with graphene composites have mitigated the main drawbacks of photocatalytic disinfection, i.e., limited activity, which is conditioned by the lack of visible light activity and the rapid re-combination of photocatalyst’s charge carriers, but most of these studies have not been carried out for real applications. Recently, there has been great interest on visible light-active materials, focusing especially on electric and photonic properties [8]. This cluster is far away from the rest, and it presents very limited collaborations with the other topics. This is clearly seen in Figure 4a, where the red and yellow/green clusters are far apart, pointing out the need to work together to connect the world of nanomaterials to the real-world applications. In addition, this area of research has other potential applications in air purification and the sterilization of surfaces in healthcare environments.

#### 3.4.7. Keywords’ Trends and Progress

Figure 4b shows that the keywords colored in green to yellow (intermediate to high scores, which means that those keywords are frequent in recent years) belong mostly to the red cluster (Figure 4a), which shows that the current trend is to focus more on solar photocatalysis with TiO_2_ and nanomaterials. Recent progress has been made in catalyst modification research to boost its light-harvesting capacity towards the visible; new Vis-light active catalytic materials for disinfection have been tested, including plasmonic [60] and carbon nitride-based photocatalysts [61]. There have also been advances in disinfection with TiO_2_, based on the applications of its different types of structures (nanotubes, nanoparticles, nanofibers or nanopowder) doped with silver, selenium nanoparticles, ZnO, copper, zinc or yttrium, and for several waterborne microorganisms such as *Escherichia* *coli, Staphylococcus aureus,* Bacteriophages F2 and MS2, *Cryptosporidium spp.* and *Candida albicans* [62].

One area of growing interest (shown in green in Figure 4b) relates to *antimicrobial-resistant bacteria* (ARB). According to the WHO, the mitigation of antimicrobial resistance will have a major impact on SDG6, estimating that the health costs will be 1.2 billion dollars by 2050 owing to increased antimicrobial resistance (https://www.who.int/health-topics/antimicrobial-resistance, accessed on 9 June 2021). This issue becomes particularly serious when pathogenic bacteria carrying antibiotic-resistant genes (ARG) re-grow after disinfection treatments are applied to WWTP effluents. In the recent years, due to the high consumption of antibiotics, ARB and ARG have been increasingly found and monitored in waters of different origins such as municipal and hospital WW, surface water and groundwater, sediments, water used in agriculture, drinking water, and even in the air, crops, and soils [43,63]. Various disinfection methods based on sunlight or artificial sunlight, such as UV/solar/H_2_O_2_, Fenton and photo-Fenton, and heterogeneous catalysis with TiO_2_, have been tested for treating numerous ARG and ARB [27]; however, although the majority of AOPs have yielded very high levels of inactivation, further investigation is still needed to find solutions to secondary contamination, for example, the release and transfer of ARG among microbial cells, photo-reactivation, etc. [63], which imply the use of post-treatments following AOP [64].

Figure 4a offers a view of the research work that has been carried out in the field, while Figure 4b gives an idea of the past and present trends over the last decade. The joint analysis of Figure 4a,b provides the research trends and gaps.

## 4. Conclusions

### 4.1. General Remarks

Revising the research questions proposed at the introduction of this bibliometric study, we have found that disinfection mediated by solar radiation is a research area of growing interest, the USA being the country with the most publications, although distributed among many centers, while research in Europe focuses on well-established groups from Spain, Switzerland, Ireland, and United Kingdom. In addition, high-impact factor journals in the area of water, and similar, stand out in this field, which gives an idea of the great impact of this research topic. Finally, from the analysis of the keywords the following conclusions are made:

- The studies of disinfection mediated by sunlight focus mainly on water for human consumption and water for reuse (e.g., in crop irrigation), which may come from WWTPs.
- The research done shows that the organic matter present in the water has a great influence on the level of disinfection achieved.
- Recent pilot-scale research has shown the great potential of solar technologies for commercial scale-up.
- The evolution of the keywords in the last decade indicates that solar photocatalysis with new materials that accelerate oxidative reactions is the area most studied in recent years.

### 4.2. Prospects and Future Research

Several research gaps that require further investigation have been clearly identified by the analysis made in this study:

- Better and deeper assessment of whether the level of disinfection is acceptable for drinking or for the selected restricted reuse (e.g., irrigation, environmental, aquifers’ recharge, etc.).
- Further analysis of the impact on public health when a drinking water intervention is undertaken and demonstrating impact in terms of risk reduction in food production when treated WW is reused for this purpose. In this sense, it is very advisable to appropriately assess bacterial/viral regrowth and evaluate microorganisms’ post-treatment recovery capacity to guarantee the positive impacts in health, food security and environment of the disinfection treatment in the overall process.
- More testing of solar treatments for natural waters or with consortia of microorganisms naturally present in the waters to evaluate the potential of the real-world application of the technology.
- Focusing not only on the assessment of regulated microorganisms’ indicators, but also of new emerging pathogens (e.g., ARB) to corroborate that the technology reaches the minimum requirements of water quality or, otherwise, which post-treatments are required to achieve water quality standards.
- Considering the large-scale application, the investment and maintenance of the solar hardware, and the life span of the materials to give realistic figures about economic sustainability of the technology and to identity limitations or new research needs. Furthermore, field trials are needed to identify local limitations and to optimize the technology for the different areas.
- Solving the great limitation of solar photocatalysis with new materials for realistic drinking water or WW disinfection applications.

Considering that access to water is a human right, it might be strongly necessary to take into account the human right-based approach of this area. Therefore, the key criteria to select water technologies that accommodate and assist our needs from a global point of view might guarantee the availability, quality, acceptability, accessibility, and affordability of water for all, including gender balance, in line with the Goal 6 of the UN-2030 agenda.

## Figures and Tables

**Table 1:** Principal events, breakthroughs, and contributors for solar water disinfection (SODIS) (above) and water disinfection by solar photocatalysis (below).

**SODIS**	
**1980s**	A. Acra et al., noted that sunlight destroyed bacteria in contaminated water, including pathogens, and they investigated the applicability of this method to the disinfection of oral rehydration solutions prepared with the contaminated water [31].
**1990s**	Several research groups started analyzing SODIS efficiency in batch and continuous reactors, at different temperatures, radiation intensities, UV-A doses, etc. [32].
**2000–2020**	Emergency water treatment following natural disasters and during humanitarian crises was recognized by the WHO and UNICEF as of 2005 [10]. Larger water containers were successfully tested [10,16,33]. New emerging and resistant pathogens were inactivated showing that SODIS was effective against almost all waterborne microbial species of pathogenic interest [10,34]. Water turbidity was overcome [34]. Chemical additives were used as possible enhancers [10]. Models and mechanisms of solar radiation induced cell death in water were studied [35]. Studies were carried out on possible leach of unsafe chemicals from the plastic bottles [34]. Enhancement technologies (flow reactors, continuous reactors, solar mirrors, etc.) [10,34].
**Water disinfection by solar photocatalysis**	
**1980s**	The work of Matsunaga and coworkers (1985) was the first report of photocatalytic disinfection (TiO_2_) [36].
**1990s**	Butterfield and coworkers showed the first study on immobilization of the catalyst [37].
**2000–2020**	Different types of structures of TiO_2_ and TiO_2_-doped nanomaterials for visible light activity [20]. First trials at circumneutral pH with photo-Fenton [38]. Development of new visible light-active catalysts [8]. New reactor design [39].

**Table 2:** Affiliation (sorted alphabetically) and authors with publications on solar-driven disinfection. In the case of authors, only the most prolific and affiliated to centers that have published more than 10 documents are listed. N_c_: Documents by institution (not per country), and N_a_: Documents by author (only one or two of the most prolific authors are listed per center). H: H-index.

Center/University	Country	N_c_	Author/s	N_a_	H
CIEMAT-Plataforma Solar de Almería	Spain	152	Polo-López, M. I. Malato, S.	42 22	31 81
Eawag - Swiss Federal Institute of Aquatic Science and Technology	Switzerland	36	Mosler, H. J.	14	23
Ecole Polytechnique Fédérale de Lausanne	Switzerland	79	Pulgarín, C. Giannakis, S. *	64 35	65 25
Royal College of Surgeons in Ireland RCSI	Ireland	45	McGuigan, K. Conroy, R. M.	43 12	31 54
Ministry of Education China	China	28	Li, Y.	3	24
Ulster University	United Kingdom	31	Fernández Ibáñez, P. ** Byrne, J. P.	77 13	52 33
Universidad de Almeria	Spain	34	Sánchez Pérez J. A.	11	45
Universidad Rey Juan Carlos	Spain	17	Marugán, J.	14	29
University of California, Berkeley	United States	18	Nelson, K. L.	7	39

* Currently researching at the Polytechnic University of Madrid. ** Currently at Ulster University, although her previous affiliation was CIEMAT.

## Data Availability

Not applicable.

## Supplementary Materials

The following are available online at https://www.mdpi.com/article/10.3390/ijerph18179396/s1, Figure S1: Types of documents in the field of research of solar-driven water disinfection from 1977 to 2020; Figure S2: Evolution of the percentages of documents published Open Access and others over the last 10 years in the field of solar-driven water disinfection; Figure S3: Relationships between the different countries that collaborate with joint publications on solar-driven water disinfection; Table S1: Funding sponsor of publications on solar-driven water disinfection; File S1: Data matrix: Search export from Scopus database.

## References

[1] Drinking-Water. https://www.who.int/news-room/fact-sheets/detail/drinking-water.

[2] United Nations Resolution adopted by the General Assembly A/RES/70/1. Transforming our world: The 2030 Agenda for Sustainable Development 2015. https://www.un.org/en/development/desa/population/migration/generalassembly/docs/globalcompact/A_RES_70_1_E.pdf.

[3] World Health Organization (2019). Results of Round II of the WHO International Scheme to Evaluate Household Water Treatment Technologies.

[4] (2015). UNESCO ODS6. https://www.un.org/sustainabledevelopment/es/water-and-sanitation/.

[5] Richardson S., Plewa M., Wagner E., Schoeny R., Demarini D. (2007). Occurrence, Genotoxicity, and Carcinogenicity of Regulated and Emerging Disinfection by-Products in Drinking Water: A Review and Roadmap for Research. Mutat. Res. Mutat. Res..

[6] Lei X., Lei Y., Zhang X., Yang X. (2021). Treating Disinfection Byproducts with UV or Solar Irradiation and in UV Advanced Oxidation Processes: A Review. J. Hazard. Mater..

[7] Wang M., Ateia M., Awfa D., Yoshimura C. (2021). Regrowth of Bacteria after Light-Based Disinfection—What We Know and Where We Go from Here. Chemosphere.

[8] Pelaez M., Nolan N.T., Pillai S.C., Seery M.K., Falaras P., Kontos A.G., Dunlop P.S.M., Hamilton J.W.J., Byrne J.A., O’Shea K. (2012). A Review on the Visible Light Active Titanium Dioxide Photocatalysts for Environmental Applications. Appl. Catal. B Environ..

[9] Byrne J.A., Fernandez-Ibañez P.A., Dunlop P.S.M., Alrousan D.M.A., Hamilton J.W.J. (2011). Photocatalytic Enhancement for Solar Disinfection of Water: A Review. Int. J. Photoenergy.

[10] McGuigan K.G., Conroy R.M., Mosler H.-J., du Preez M., Ubomba-Jaswa E., Fernandez-Ibañez P. (2012). Solar Water Disinfection (SODIS): A Review from Bench-Top to Roof-Top. J. Hazard. Mater..

[11] Polo-López M.I., Martínez-García A., Abeledo-Lameiro M.J., Gómez-Couso H.H., Ares-Mazás E.E., Reboredo-Fernández A., Morse T.D., Buck L., Lungu K., McGuigan K.G. (2019). Microbiological Evaluation of 5 L- and 20 L-Transparent Polypropylene Buckets for Solar Water Disinfection (SODIS). Molecules.

[12] Sansaniwal S.K. (2019). Advances and Challenges in Solar-Powered Wastewater Treatment Technologies for Sustainable Development: A Comprehensive Review. Int. J. Ambient Energy.

[13] Sobsey M.D., World Health Organization (2002). Water, Sanitation and Health Team. Managing Water in the Home: Accelerated Health Gains from Improved Water Supply/Prepared by Mark D. Sobsey.

[14] Danwittayakul S., Songngam S., Fhulua T., Muangkasem P., Sukkasi S. (2017). Safety and Durability of Low-Density Polyethylene Bags in Solar Water Disinfection Applications. Environ. Technol..

[15] Ubomba-Jaswa E., Fernández-Ibáñez P., McGuigan K.G. (2010). A Preliminary Ames Fluctuation Assay Assessment of the Genotoxicity of Drinking Water That Has Been Solar Disinfected in Polyethylene Terephthalate (PET) Bottles. J. Water Health.

[16] Martínez-García A., Vincent M., Rubiolo V., Domingos M., Canela M.C., Oller I., Fernández-Ibáñez P., Polo-López M.I. (2020). Assessment of a Pilot Solar V-Trough Reactor for Solar Water Disinfection. Chem. Eng. J..

[17] Reyneke B., Ndlovu T., Vincent M.B., Martínez-García A., Polo-López M.I., Fernández-Ibáñez P., Ferrero G., Khan S., McGuigan K.G., Khan W. (2020). Validation of Large-Volume Batch Solar Reactors for the Treatment of Rainwater in Field Trials in Sub-Saharan Africa. Sci. Total Environ..

[18] Polo-López M.I., Fernández-Ibáñez P., Ubomba-Jaswa E., Navntoft C., García-Fernández I., Dunlop P.S.M., Schmid M., Byrne J.A., McGuigan K.G. (2011). Elimination of Water Pathogens with Solar Radiation Using an Automated Sequential Batch CPC Reactor. J. Hazard. Mater..

[19] Malato S., Fernández-Ibáñez P., Maldonado M.I., Blanco J., Gernjak W. (2009). Decontamination and Disinfection of Water by Solar Photocatalysis: Recent Overview and Trends. Catal. Today.

[20] Fagan R., McCormack D.E., Dionysiou D.D., Pillai S.C. (2016). A Review of Solar and Visible Light Active TiO_2_ Photocatalysis for Treating Bacteria, Cyanotoxins and Contaminants of Emerging Concern. Mater. Sci. Semicond. Process..

[21] Dalrymple O.K., Stefanakos E., Trotz M.A., Goswami D.Y. (2010). A Review of the Mechanisms and Modeling of Photocatalytic Disinfection. Appl. Catal. B Environ..

[22] Giannakis S., Polo López M.I., Spuhler D., Sánchez Pérez J.A., Fernández Ibáñez P., Pulgarin C. (2016). Solar Disinfection Is an Augmentable, in Situ-Generated Photo-Fenton Reaction—Part 1: A Review of the Mechanisms and the Fundamental Aspects of the Process. Appl. Catal. B Environ..

[23] Polo-López M.I., Oller I., Fernández-Ibáñez P. (2013). Benefits of Photo-Fenton at Low Concentrations for Solar Disinfection of Distilled Water. A Case Study: Phytophthora Capsici. Catal. Today.

[24] Rodríguez-Chueca J., Polo-López M.I., Mosteo R., Ormad M.P., Fernández-Ibáñez P. (2014). Disinfection of Real and Simulated Urban Wastewater Effluents Using a Mild Solar Photo-Fenton. Appl. Catal. B Environ..

[25] Ortega-Gómez E., Ballesteros Martín M.M., Carratalà A., Fernández Ibañez P., Sánchez Pérez J.A., Pulgarín C. (2015). Principal Parameters Affecting Virus Inactivation by the Solar Photo-Fenton Process at Neutral PH and ΜM Concentrations of H_2_O_2_ and Fe^2+/3+^. Appl. Catal. B Environ..

[26] Ruiz-Aguirre A., Polo-López M.I., Fernández-Ibáñez P., Zaragoza G. (2017). Integration of Membrane Distillation with Solar Photo-Fenton for Purification of Water Contaminated with *Bacillus* sp. and *Clostridium* sp. Spores. Sci. Total Environ..

[27] Moreira N.F.F., Narciso-da-Rocha C., Polo-López M.I., Pastrana-Martínez L.M., Faria J.L., Manaia C.M., Fernández-Ibáñez P., Nunes O.C., Silva A.M.T. (2018). Solar Treatment (H_2_O_2_, TiO_2_-P_25_ and GO-TiO_2_ Photocatalysis, Photo-Fenton) of Organic Micropollutants, Human Pathogen Indicators, Antibiotic Resistant Bacteria and Related Genes in Urban Wastewater. Water Res..

[28] Giannakis S., López M.I.P., Spuhler D., Pérez J.A.S., Ibáñez P.F., Pulgarin C. (2016). Solar Disinfection Is an Augmentable, in Situ-Generated Photo-Fenton Reaction—Part 2: A Review of the Applications for Drinking Water and Wastewater Disinfection. Appl. Catal. B Environ..

[29] De la Obra Jiménez I., Esteban García B., Rivas Ibáñez G., Casas López J.L., Sánchez Pérez J.A. (2019). Continuous Flow Disinfection of WWTP Secondary Effluents by Solar Photo-Fenton at Neutral PH in Raceway Pond Reactors at Pilot Plant Scale. Appl. Catal. B Environ..

[30] De la Obra Jiménez I., Giannakis S., Grandjean D., Breider F., Grunauer G., Casas López J.L., Sánchez Pérez J.A., Pulgarin C. (2020). Unfolding the Action Mode of Light and Homogeneous vs. Heterogeneous Photo-Fenton in Bacteria Disinfection and Concurrent Elimination of Micropollutants in Urban Wastewater, Mediated by Iron Oxides in Raceway Pond Reactors. Appl. Catal. B Environ..

[31] Acra A., Karahagopian Y., Raffoul Z., Dajani R. (1980). Disinfection of Oral Rehydration Solutions by Sunlight. Lancet.

[32] Sommer B., Marino A., Solarte Y., Salas M.L., Dierolf C., Valiente C., Mora D., Rechsteiner R., Setter P., Wirojanagud W. (1997). SODIS—An Emerging Water Treatment Process. J. Water Supply Res. Technol. AQUA.

[33] Borde P., Elmusharaf K., McGuigan K.G., Keogh M.B. (2016). Community Challenges When Using Large Plastic Bottles for Solar Energy Disinfection of Water (SODIS). BMC Public Health.

[34] Chaúque B.J.M., Rott M.B. (2021). Solar Disinfection (SODIS) Technologies as Alternative for Large-Scale Public Drinking Water Supply: Advances and Challenges. Chemosphere.

[35] García-Gil Á., García-Muñoz R.A., McGuigan K.G., Marugán J. (2021). Solar Water Disinfection to Produce Safe Drinking Water. A Review of Parameters, Enhancements, and Modelling Approaches to Make SODIS Faster and Safer. Molecules.

[36] Matsunaga T., Tomoda R., Nakajima T., Wake H. (1985). Photoelectrochemical Sterilization of Microbial Cells by Semi-Conductor Powders. FEMS Microbiol. Lett..

[37] Butterfield I.M., Christensen P.A., Curtis T.P., Gunlazuardi J. (1997). Water Disinfection Using an Immobilised Titanium Dioxide Film in a Photochemical Reactor with Electric Field Enhancement. Water Res..

[38] Rincón A.G., Pulgarin C. (2006). Comparative Evaluation of Fe^3+^ and TiO_2_ Photoassisted Processes in Solar Photocatalytic Disinfection of Water. Appl. Catal. B Environ..

[39] Keane D.A., McGuigan K.G., Ibáñez P.F., Polo-Lopez M.I., Byrne J.A., Dunlop P.S.M., O’Shea K., Dionysiou D.D., Pillai S.C. (2014). Solar Photocatalysis for Water Disinfection: Materials and Reactor Design. Catal. Sci. Technol..

[40] Mongeon P., Paul-Hus A. (2016). The Journal Coverage of Web of Science and Scopus: A Comparative Analysis. Scientometrics.

[41] Martín-Martín A., Orduna-Malea E., Thelwall M., Delgado López-Cózar E. (2018). Google Scholar, Web of Science, and Scopus: A Systematic Comparison of Citations in 252 Subject Categories. J. Informetr..

[42] Van Eck N.J., Waltman L. (2010). Software Survey: VOSviewer, a Computer Program for Bibliometric Mapping. Scientometrics.

[43] Ferro G., Polo-López M.I., Martínez-Piernas A.B., Fernández-Ibáñez P., Agüera A., Rizzo L. (2015). Cross-Contamination of Residual Emerging Contaminants and Antibiotic Resistant Bacteria in Lettuce Crops and Soil Irrigated with Wastewater Treated by Sunlight/H_2_O_2_. Environ. Sci. Technol..

[44] Aguas Y., Hincapie M., Martínez-Piernas A.B., Agüera A., Fernández-Ibáñez P., Nahim-Granados S., Polo-López M.I. (2019). Reclamation of Real Urban Wastewater Using Solar Advanced Oxidation Processes: An Assessment of Microbial Pathogens and 74 Organic Microcontaminants Uptake in Lettuce and Radish. Environ. Sci. Technol..

[45] Becerra-Castro C., Lopes A.R., Vaz-Moreira I., Silva E.F., Manaia C.M., Nunes O.C. (2015). Wastewater Reuse in Irrigation: A Microbiological Perspective on Implications in Soil Fertility and Human and Environmental Health. Environ. Int..

[46] Rincón A., Pulgarín C. (2004). Effect of PH, Inorganic Ions, Organic Matter and H_2_O_2_ on *E*. coli K12 Photocatalytic Inactivation by TiO2Implications in Solar Water Disinfection. Appl. Catal. B Environ..

[47] Ortega-Gómez E., Ballesteros Martín M.M., Esteban García B., Sánchez Pérez J.A., Fernández Ibáñez P. (2014). Solar Photo-Fenton for Water Disinfection: An Investigation of the Competitive Role of Model Organic Matter for Oxidative Species. Appl. Catal. B Environ..

[48] Rommozzi E., Giannakis S., Giovannetti R., Vione D., Pulgarin C. (2020). Detrimental vs. Beneficial Influence of Ions during Solar (SODIS) and Photo-Fenton Disinfection of E. Coli in Water: (Bi)Carbonate, Chloride, Nitrate and Nitrite Effects. Appl. Catal. B Environ..

[49] Zhang C., Li Y., Shuai D., Shen Y., Wang D. (2019). Progress and Challenges in Photocatalytic Disinfection of Waterborne Viruses: A Review to Fill Current Knowledge Gaps. Chem. Eng. J..

[50] Roshith M., Pathak A., Nanda Kumar A.K., Anantharaj G., Saranyan V., Ramasubramanian S., Satheesh Babu T.G., Ravi Kumar D.V. (2021). Continuous Flow Solar Photocatalytic Disinfection of *E*. coli Using Red Phosphorus Immobilized Capillaries as Optofluidic Reactors. Appl. Surf. Sci..

[51] Chaúque B.J.M., Benetti A.D., Corção G., Silva C.E., Gonçalves R.F., Rott M.B. (2021). A New Continuous-Flow Solar Water Disinfection System Inactivating Cysts of Acanthamoeba Castellanii, and Bacteria. Photochem. Photobiol. Sci..

[52] Clasen T., Haller L., Walker D., Bartram J., Cairncross S. (2007). Cost-Effectiveness of Water Quality Interventions for Preventing Diarrhoeal Disease in Developing Countries. J. Water Health.

[53] Nahim-Granados S., Rivas-Ibáñez G., Antonio Sánchez Pérez J., Oller I., Malato S., Polo-López M.I. (2020). Fresh-Cut Wastewater Reclamation: Techno-Economical Assessment of Solar Driven Processes at Pilot Plant Scale. Appl. Catal. B Environ..

[54] Burgess T. WaterAid. 2016. Water at What Cost? The State of the World’s Water 2016. Briefing Report. https://washmatters.wateraid.org/sites/g/files/jkxoof256/files/Water%20%20At%20What%20Cost%20%20The%20State%20of%20the%20Worlds%20Water%202016.pdf.

[55] Morse T., Luwe K., Lungu K., Chiwaula L., Mulwafu W., Buck L., Harlow R., Fagan G.H., McGuigan K. (2020). A Transdisciplinary Methodology for Introducing Solar Water Disinfection to Rural Communities in Malawi—Formative Research Findings. Integr. Environ. Assess. Manag..

[56] Krafft M.P., Riess J.G. (2015). Per- and Polyfluorinated Substances (PFASs): Environmental Challenges. Curr. Opin. Colloid Interface Sci..

[57] Méndez-Hermida F., Ares-Mazás E., McGuigan K.G., Boyle M., Sichel C., Fernández-Ibáñez P. (2007). Disinfection of Drinking Water Contaminated with Cryptosporidium Parvum Oocysts under Natural Sunlight and Using the Photocatalyst TiO_2_. J. Photochem. Photobiol. B.

[58] Abeledo-Lameiro M.J., Polo-López M.I., Ares-Mazás E., Gómez-Couso H. (2019). Inactivation of the Waterborne Pathogen Cryptosporidium Parvum by Photo-Fenton Process under Natural Solar Conditions. Appl. Catal. B Environ..

[59] Castro-Alférez M., Polo-López M.I., Marugán J., Fernández-Ibáñez P. (2017). Mechanistic Modeling of UV and Mild-Heat Synergistic Effect on Solar Water Disinfection. Chem. Eng. J..

[60] Endo-Kimura M., Kowalska E. (2020). Plasmonic Photocatalysts for Microbiological Applications. Catalysts.

[61] Wang W., Zhou C., Yang Y., Zeng G., Zhang C., Zhou Y., Yang J., Huang D., Wang H., Xiong W. (2021). Carbon Nitride Based Photocatalysts for Solar Photocatalytic Disinfection, Can We Go Further?. Chem. Eng. J..

[62] Gopinath K.P., Madhav N.V., Krishnan A., Malolan R., Rangarajan G. (2020). Present Applications of Titanium Dioxide for the Photocatalytic Removal of Pollutants from Water: A Review. J. Environ. Manag..

[63] Anthony E.T., Ojemaye M.O., Okoh O.O., Okoh A.I. (2020). A Critical Review on the Occurrence of Resistomes in the Environment and Their Removal from Wastewater Using Apposite Treatment Technologies: Limitations, Successes and Future Improvement. Environ. Pollut..

[64] Michael S.G., Michael-Kordatou I., Beretsou V.G., Jäger T., Michael C., Schwartz T., Fatta-Kassinos D. (2019). Solar Photo-Fenton Oxidation Followed by Adsorption on Activated Carbon for the Minimisation of Antibiotic Resistance Determinants and Toxicity Present in Urban Wastewater. Appl. Catal. B Environ..

