# Supplementary material for: Worldwide Research Trends on Solar-Driven Water Disinfection
Source: Int J Environ Res Public Health. 2021 Sep 6;18(17):9396. doi: 10.3390/ijerph18179396 (PMC8430867; doi:10.3390/ijerph18179396)
Supplement: Supplementary file 1 [file ijerph-18-09396-s001.zip › ijerph-1364612-supplementary/Supplementary files/Supplementary.pdf]

SUPPORTING INFORMATION

WORLDWIDE RESEARCH TRENDS ON SOLAR-DRIVEN WATER DISINFECTION

Ballesteros Martín MM[a,*], Brindley C[b], Sánchez Pérez JA[b,c] and Fernández-Ibañez P[d**]

[a,*]Molecular Biology and Biochemical Engineering Dpt., Experimental Sciences Faculty, Pablo de Olavide University, Ctra. de Utrera km 1, Seville, 41013, Spain

[b]Department of Chemical Engineering, University of Almería, Almería, Spain

[c]CIESOL, Joint Centre of the University of Almería-CIEMAT, Almería, 04120, Spain

[d,**]Nanotechnology and Integrated BioEngineering Centre, School of Engineering, Ulster University, Northern Ireland, BT37 0QB, United Kingdom

*Corresponding author: e-mail: mmbalmar@upo.es

**Corresponding author: e-mail: p.fernandez@ulster.ac.uk

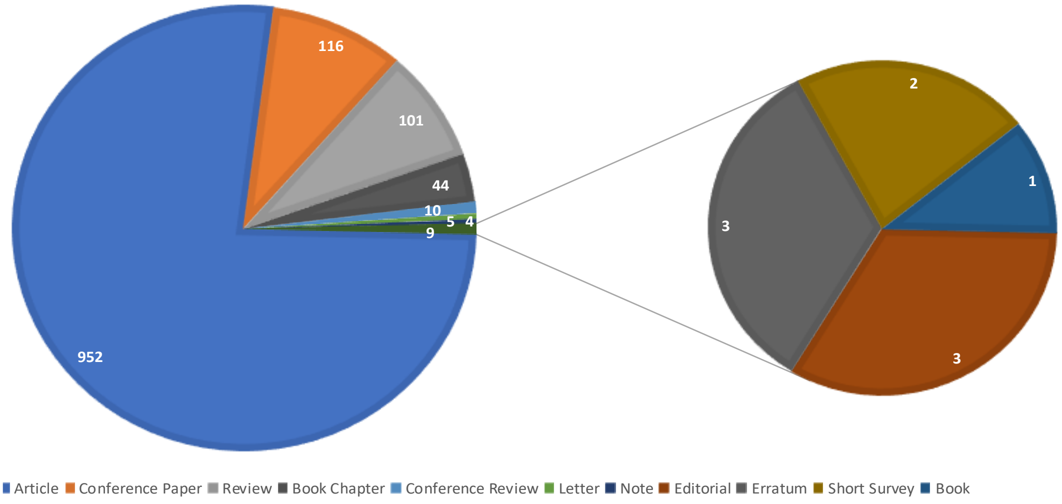

Figure S1: Types of documents in the field of research of solar-driven water disinfection from 1977 to 2020.

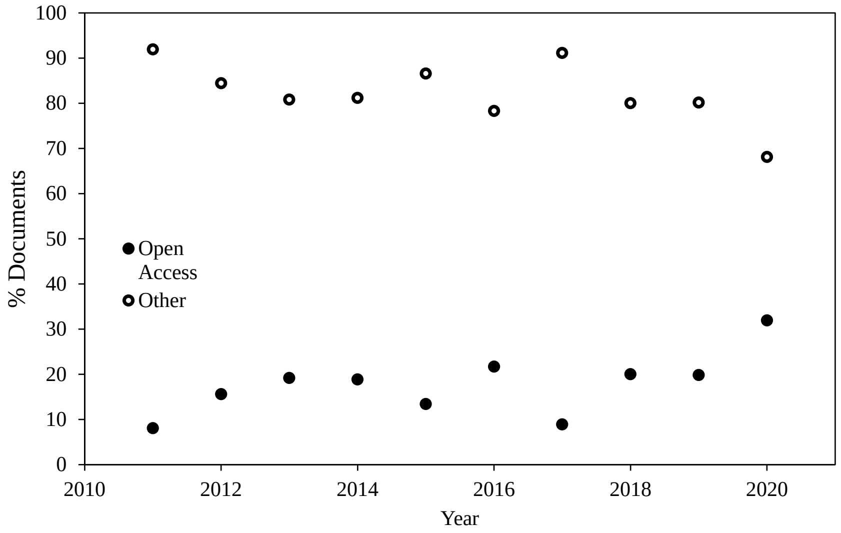

Figure S2: Evolution of the percentages of documents published Open Access and other over the last 10 years in the field of solar-driven water disinfection.

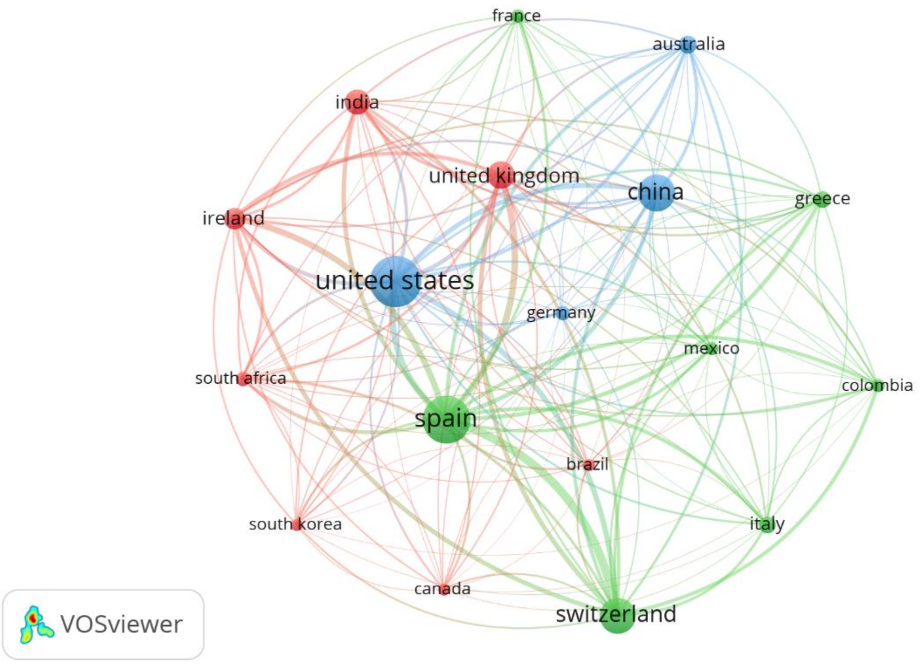

Figure S3: Relationships between the different countries that collaborate with joint publications on solar-driven water disinfection.

Table S1: Funding sponsor of publications on solar-driven water disinfection.

**Sponsor**	**Country**	**Documents**
National Natural Science Foundation of China	China	73
European Commission	Europe	47
National Science Foundation	United States	29
European Regional Development Fund	Europe	20
Fundamental Research Funds for the Central Universities	China	15
Ministerio de Ciencia e Innovación	Spain	15
Direktionfür Entwicklung und Zusammenarbeit	Switzerland	12
Horizon 2020 Framework Programme	Europe	12
Ministerio de Economía y Competitividad	Spain	11
Schweizerische Nationalfondszur Förderung der wissenschaftlichen Forschung	Switzerland	11
